# A comparison of three clustering methods for finding subgroups in MRI, SMS or clinical data: SPSS TwoStep Cluster analysis, Latent Gold and SNOB

**DOI:** 10.1186/1471-2288-14-113

**Published:** 2014-10-02

**Authors:** Peter Kent, Rikke K Jensen, Alice Kongsted

**Affiliations:** School of Sports Science and Clinical Biomechanics, University of Southern Denmark, Campusvej 55, Odense, M 5230 Denmark; Research Department, Spine Centre of Southern Denmark, Hospital Lillebaelt, Institute of Regional Health Services Research, University of Southern Denmark, Middelfart, Denmark; Nordic Institute of Chiropractic and Clinical Biomechanics, University of Southern Denmark, Odense, Denmark

**Keywords:** Cluster analysis, Latent Class Analysis, Head-to-head comparison, Reproducibility, MRI, SMS

## Abstract

**Background:**

There are various methodological approaches to identifying clinically important subgroups and one method is to identify clusters of characteristics that differentiate people in cross-sectional and/or longitudinal data using Cluster Analysis (CA) or Latent Class Analysis (LCA). There is a scarcity of head-to-head comparisons that can inform the choice of which clustering method might be suitable for particular clinical datasets and research questions. Therefore, the aim of this study was to perform a head-to-head comparison of three commonly available methods (SPSS TwoStep CA, Latent Gold LCA and SNOB LCA).

**Methods:**

The performance of these three methods was compared: (i) quantitatively using the number of subgroups detected, the classification probability of individuals into subgroups, the reproducibility of results, and (ii) qualitatively using subjective judgments about each program’s ease of use and interpretability of the presentation of results.

We analysed five real datasets of varying complexity in a secondary analysis of data from other research projects. Three datasets contained only MRI findings (n = 2,060 to 20,810 vertebral disc levels), one dataset contained only pain intensity data collected for 52 weeks by text (SMS) messaging (n = 1,121 people), and the last dataset contained a range of clinical variables measured in low back pain patients (n = 543 people). Four artificial datasets (n = 1,000 each) containing subgroups of varying complexity were also analysed testing the ability of these clustering methods to detect subgroups and correctly classify individuals when subgroup membership was known.

**Results:**

The results from the real clinical datasets indicated that the number of subgroups detected varied, the certainty of classifying individuals into those subgroups varied, the findings had perfect reproducibility, some programs were easier to use and the interpretability of the presentation of their findings also varied. The results from the artificial datasets indicated that all three clustering methods showed a near-perfect ability to detect known subgroups and correctly classify individuals into those subgroups.

**Conclusions:**

Our subjective judgement was that Latent Gold offered the best balance of sensitivity to subgroups, ease of use and presentation of results with these datasets but we recognise that different clustering methods may suit other types of data and clinical research questions.

## Background

There is increasing interest in the identification of clinically important patient subgroups in order to better target treatment, make more accurate estimates of prognosis, and improve health system efficiency by providing the right treatment to the right patient at the right time [1, 2]. This is especially so in non-specific health conditions that are highly prevalent, costly and have a high burden of disease. For example, most back pain is non-specific and yet it is the leading cause of disability globally [3]. Identifying subgroups of findings can also be useful in imaging data, such as Magnetic Resonance Imaging (MRI) findings [4, 5] and longitudinal data describing clinical or life course trajectories [6]. Longitudinal data may be collected using many methods but an increasingly used method is via Short Message Service (SMS) text messaging [7].

There are various methodological approaches to identifying subgroups, although the same validation stages are required before clinical importance can be established [8, 9]. Some statistical approaches to subgrouping work backwards from an outcome, such as using good response to a treatment, as a way to identify the clinical characteristics of people most likely to respond to that therapy [10]. Other statistical methods seek to identify clusters of symptoms and signs that differentiate people, in cross-sectional and/or longitudinal data. This approach was taken by Beneciuk et al. [11], who used cluster analysis of baseline fear avoidance data from patients in a clinical trial and found three distinct subgroups (low risk, high specific fear, and high fear and catastrophising) that were associated with different clinical trajectories.

Historically, cluster analysis methods (hierarchical or k-means clustering) have been used but more recently these have been complemented by probabilistic (Bayesian) methods, such as Latent Class Analysis (LCA). Traditional cluster analysis methods initially create a distance measure of dissimilarity between individuals (such as a Euclidean distance), and then seek to determine the underlying subgroup structure by optimising the within-subgroup variability of individuals’ distance measures and maximising their between group variability. In contrast, LCA methods initially use a probabilistic modeling approach (such as finite mixture modeling) to identify the likely distributions with the data and the likely placement of individuals within those distributions. They then seek to determine the optimal subgroup structure that explains the most variance while requiring the simplest specification of the model (the optimal balance between the most explanatory models and parsimonious models). In this study we use the term ‘clustering methods’ as an umbrella term to cover both ‘distance-based cluster analysis’ approaches and ‘probability-based LCA’ approaches.

LCA has a number of advantages, including being able: to better manage variables of mixed measurement types (dichotomous, ordinal, interval scales and scales of varying width), to better handle missing data, to provide classification probabilities for individual classification, to provide model-based parameters that can be used to classify new individuals not in the derivation sample, and to have greater classification accuracy [12–14]. LCA methods are now easily accessible to clinical researchers and the use of these computationally-intense software programs has been facilitated by the speed of contemporary computers.

There are many computer programs available for LCA but there is a scarcity of head-to-head comparisons published that can inform the choice of which LCA might be suitable for particular clinical datasets and research questions. There are only two such studies that we are aware of. Haughton et al. [15] compared three LCA programs (Latent Gold, poLCA and MCLUST) using a single dataset. All three programs identified the same number of subgroups, though there was some variation in the individuals allocated to those subgroups. Those authors indicated that their results may not hold for other datasets and that the use of poLCA and MCLUST require proficiency in the R programming environment and language. Bacher et al. [12] compared a distance-based cluster method (SPSS TwoStep), and two LCA methods (Latent Gold and ALMO) using five artificial datasets with known subgroups. TwoStep is a hybrid approach that uses a distance measure to separate individuals but uses a similar method to LCA to choose the optimal subgroup model, and it has been shown to perform consistently better than traditional hierarchical cluster techniques [13]. Bacher et al. found that TwoStep was least able and Latent Gold most able to detect the correct number of subgroups. In particular, TwoStep had difficulties when the dataset contained a mix of nominal and interval data.

However, there are other LCA methods readily available, other measures of technical performance and other more user-focused aspects for comparison. Furthermore, the performance of clustering methods can vary depending on the type of data being analysed [13] and most previous comparison studies have been written for a statistical audience rather than for clinical researchers.

Therefore the aim of this study was to perform, using a variety of types of clinical and artificial datasets, a head-to-head comparison of three commonly available clustering methods (TwoStep, Latent Gold and SNOB), based on the evaluation criteria of: the number of subgroups detected, the classification probability of individuals to those subgroups, the reproducibility of the findings, and each computer program’s ease of use and interpretability of the presentation of results. These evaluation criteria were orientated towards informing the decisions of clinical researchers, rather than statisticians, and therefore clinical rather than mathematical language is used and clinical considerations are emphasised.

## Method

### Clustering software

This study investigated the use of three clustering methods, each implemented within a separate software program: (i) TwoStep Cluster Analysis in IBM SPSS *(version 19, SPSS Statistics/IBM Corp, Chicago IL, USA)*, which is available in the base package of this program (TwoStep) [16], (ii) Latent Class Modeling in Latent Gold *(version 4.5, Statistical Innovations, Belmont MA, USA),* which is the simplest of three LCA approaches available in this program (Latent Gold) [17], and (iii) ‘vanilla’ SNOB *(version 1.15, Monash University, Melbourne, Australia),* which is the most straightforward form of this program (SNOB) [18–20] *.* SNOB is playfully named for its ability to detect classes (subgroups) and uses the Minimum Message Length principle and finite mixture modeling to probabilistically identify latent classes.

These three clustering methods were tested using their software default settings. In the case of TwoStep, this was a log-likelihood distance measure. Clustering methods, when in exploratory mode, require some form of ‘stopping rule’ to allow determination of the optimal number of subgroups. LCA methods typically include rules designed to find the subgroup solution that explains the most variance while requiring the simplest specification of the model. Examples of such rules are the Schwarz’s Bayesian Information Criterion (BIC), Akaike’s Information Criterion, and Minimum Message Length principle. In the case of TwoStep, there is a choice of BIC or Akaike’s Information Criterion, with a default setting of BIC, and the program automatically determines the optimal solution based on the chosen criterion. Latent Gold requires the analyst to choose the optimal model and provides a number of criteria that can be used to inform that choice, the most commonly used single criterion being BIC. When using Latent Gold, we increased the number of investigated clusters until BIC did not decrease any further and chose the subgroup model with the lowest BIC and fewest subgroups. SNOB uses only Minimum Message Length and fully automates the choice of model.

### Real data sets

We analysed five datasets of varying size, type and complexity. All were a secondary analysis of real data collected for other research projects. Three datasets (MRI^1^, MRI^2^, MRI^3^) contained only MRI findings (dichotomous scales), one dataset (SMS) contained only pain intensity data (0 to 10 interval scale) collected every week over a one-year period by SMS messaging, and the last dataset (clinical) contained a range of clinical variables (dichotomous, ordinal and continuous scales) measured in low back pain (LBP) patients. These datasets were purposefully chosen from those available in our research group to investigate whether the performance of these clustering programs was consistent across data size, type and complexity, as these characteristics can affect cluster models [12]. Permission was obtained from the custodians of each of these datasets for secondary use of the data within this project (Per Kjær, Rikke Kruger Jensen, Hanne Albert/Peter Kent, Alice Kongsted, Alice Kongsted, respectively).

Both Latent Gold and SNOB are able to model data in dichotomous, ordinal and continuous scales, whereas TwoStep can only model dichotomous and interval data [12]. Therefore, to be able to model data across all three clustering methods, variables in the MRI datasets that were originally coded in ordinal scales were recoded into dichotomous scales using arbitrary but clinically intuitive cut-points. The mixed data types in the clinical dataset were retained in their original formats to preserve the complexity of these data but this restricted our comparison of these data to results from Latent Gold and SNOB. An overview of the characteristics of the five data sets is presented in Table 1.

**Table 1:** **Characteristics of real datasets**

Dataset	Data type	n	Variables
MRI^1^ dataset	Dichotomous, cross-sectional data	2,060 disc levels	Disc signal intensity, loss of disc height, disc high intensity zone, location of high intensity zone, type of disc herniation, location of disc herniation, nucleus pulposus shape, annular tear anterior, annular tear posterior, annular tear right, annular tear left, location of nerve root compression, nerve root compression, anterolisthesis, retrolisthesis, top endplate defect, bottom endplate defect, Modic changes top endplate, Modic changes bottom endplate, facet joint degeneration, facet joint asymmetry, central stenosis, foraminal stenosis.
MRI^2^ dataset	Dichotomous, cross- sectional data	3,155 disc levels	Disc signal intensity, disc height, disc high intensity zone, disc contour, type of disc herniation, disc herniation signal intensity, anterolisthesis, retrolisthesis, type of endplate changes top, type of endplate changes bottom, size of endplate changes top, size of endplate changes bottom, osteophytes top, osteophytes bottom, endplate defect top, endplate defect bottom, endplate irregularity top, endplate irregularity bottom.
MRI^3^ dataset	Dichotomous, cross-sectional data	20,810 disc levels	Disc bulge, disc degeneration, disc herniation, disc high intensity zone, Modic changes Type 1, Modic changes Type 2, nerve root compression, Scheuermann's disease, spondylolisthesis, facet joint degeneration, osteoarthritis, central spinal stenosis, scoliosis, red flag condition (cancer, fracture, infection).
SMS dataset	Interval, longitudinal repeated measures data	1,121 people	Pain intensity (0 to 10) measured once a week for 52 weeks.
Clinical dataset	Mixed (dichotomous, ordinal, interval), cross-sectional data	543 people	*Dichotomous:* gender, living alone, previous episode.
			*Ordinal:* episode duration (3 categories), STarT Back Tool subgroup (3 categories).
			*Interval:* age (years), days of pain in last 2 weeks (0 to 14), Major Depression Inventory sum score (0 to 42), Fear Avoidance Beliefs Questionnaire subscale scores (physical activity 0 to 24, work 0 to 42), Coping Strategies Questionnaire subscale scores (divert attention 0 to 100, ignoring 0 to 100, praying or hoping 0 to 100, catastrophisation 0 to 100, reinterpreting 0 to 100).

All three MRI datasets were analysed at an individual vertebral disc level, where each person in the study contributed five lumbar vertebral disc levels. The MRI^1^ dataset was collected as part of the Danish ‘Backs on Funen’ longitudinal cohort study, and was taken from the baseline cohort measurement that included a lumbar MRI (n = 412 people, 2,060 disc levels). Full details of the data collection and coding have been previously reported [21]. Briefly, this cohort of people was a representative sample of the Danish general population and, who as part of the data collected in the study, had MRIs. The MRI images were quantitatively coded by an experienced musculoskeletal research radiologist using a detailed and standardised research MRI evaluation protocol that has demonstrated high reproducibility [22].

The MRI^2^ dataset is from a cohort of patients (n = 631 patients, 3,155 disc levels) who were potential participants in a randomised controlled trial [23]. The details of the data collection and coding have also been previously reported [4]. In summary, all participants were patients who had attended a Danish outpatient hospital department (the Spine Centre of Southern Denmark) from June 2006 to June 2008, where they had been referred from the primary care sector for a multidisciplinary evaluation. Potential participants were people who had LBP or leg pain of at least 3 on an 11-point Numerical Rating Scale, a duration of current symptoms from 2 to 12 months, were above 18 years of age, and who had received a lumbar MRI. The MRI images were quantitatively coded by the same research radiologist using the same MRI evaluation protocol as in the MRI^1^ dataset.

The MRI^3^ dataset was collected for a study on the prevalence of MRI-defined spinal pathologies [24] and a study of the reproducibility of coding MRI findings [25]. Full details of the data collection and coding have been reported in those studies but briefly, these data were extracted by three trained coders from the MRI reports of all people who had attended the outpatient medical department of the Spine Centre of Southern Denmark over an eight-year period (2000 to 2008) and received a lumbar spine MRI for which a narrative report could be retrieved from their electronic patient record (n = 4,162 people, 20,810 disc levels). Once trained, the inter-rater reproducibility across the 14 pathoanatomic categories for a sample of these data (n = 1,700 ratings) ranged from substantial to perfect [25]. The original MRI reports had been narrated by either of two experienced musculoskeletal radiologists.

The SMS dataset contained data on LBP intensity self-reported every week for one year by 1,121 primary care chiropractic or GP patients in Denmark. These data were collected as part of a currently unpublished cohort study designed to identify course patterns, subgroups and prognostic factors in LBP patients seeking care from general practitioners (GPs) and chiropractors. All GPs in the administrative region of Southern Denmark were invited to participate in a quality assurance program focusing on patients with LBP and the patient self-reported data used in the current study were recorded at or after the first consultation. The chiropractors were participants in a research collaboration with a clinical practice research unit that has previously been described [26]. Patient inclusion criteria were being aged 18–65 years, attending the GP or chiropractor for the first time due to the current episode of LBP, and having adequate Danish language competency. Exclusion criteria were a suspicion of inflammatory or pathological pain, and nerve root involvement requiring acute referral to surgery. The 52 weeks of pain intensity scores had a mean within-subject correlation (collinearity) over time of 0.59 (SD 0.11, full range 0.22 to 0.81). The SMS data were entered into the clustering models without reference to their time sequence, a method previously described [27].

The clinical dataset consisted of responses on an array of questionnaires from 543 people who were potential participants in a cross-sectional study of the STarT Back Screening Tool [28]. Full details of this data collection and coding have also been reported. Participants were primary care patients in 19 chiropractic clinics who were members of the same clinical practice research unit involved in the SMS dataset. Inclusion criteria were consenting people seeking care for LBP with adequate Danish literacy to understand and self-complete the questionnaire pack.

In all three clustering programs, all the variables from each dataset were simultaneously entered into the model as indicators, with no dependent, covariate or predictor variables specified. The data collection and analysis of each of the five datasets was performed with the approval of the scientific ethics committee appropriate for each study. Under Danish law, the secondary analysis of such de-identified data does not require separate ethics approval (The Act on Processing of Personal Data, December 2012, Section 5.2; Act on Research Ethics Review of Health Research Projects, October 2013, Section 14.2).

### Artificial data sets

**Figure 1:**
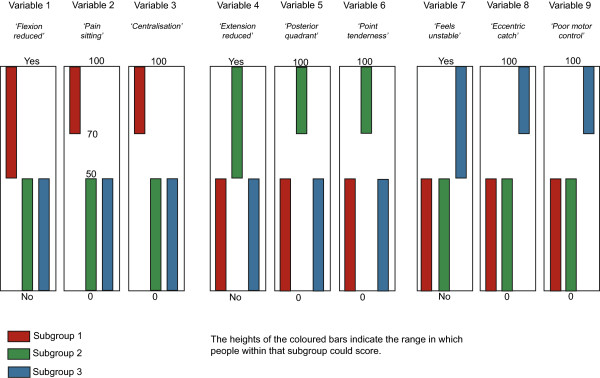
**Dataset A1 (n = 1000) - containing 3 subgroups, whose distinguishing features do not overlap, with characteristics scored on a mixture of continuous and dichotomous variables.**

**Figure 2:**
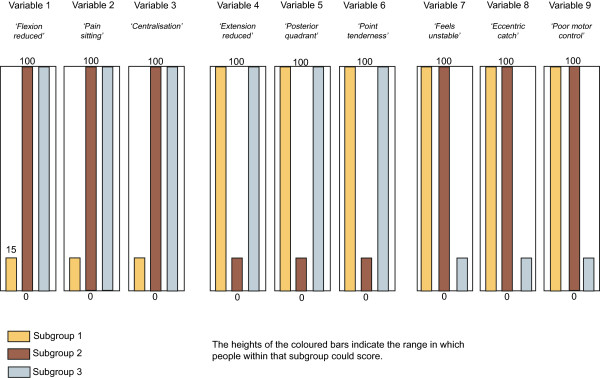
**Dataset A2 (n = 1000) - containing 3 subgroups, whose distinguishing features do overlap, with all characteristics scored on continuous variables.**

**Figure 3:**
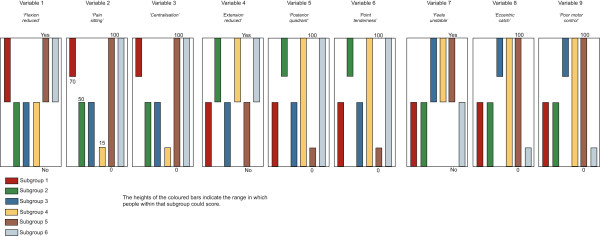
**Dataset A3 (n = 1000)- containing 6 subgroups, whose distinguishing features do overlap, with characteristics scored on a mixture of continuous and dichotomous variables.**

**Figure 4:**
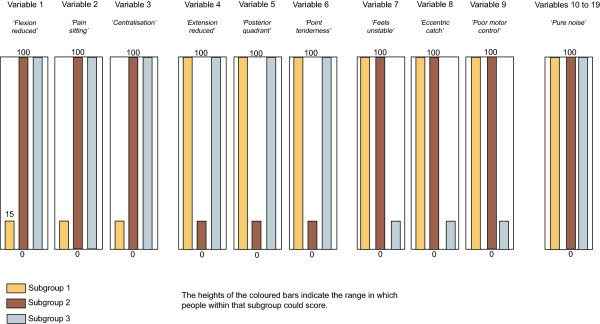
**Dataset A4 (n = 1000) - containing 3 subgroups, whose distinguishing features do overlap, with all characteristics scored on continuous variables.** Contains 10 ’pure noise’ non-discriminatory variables.

Four artificial datasets (n = 1,000 each) containing subgroups of varying complexity were created to test the ability of the clustering methods to detect subgroups and correctly classify individuals when subgroup membership was known to the researchers but withheld from the modeling process. The subgroup characteristics are described in Table 2 and illustrated in Figures 1, 2, 3 and 4. The variables were arbitrarily given clinical labels to aid comprehension but these labels were entirely fictitious.

**Table 2:** **Characteristics of artificial datasets**

Dataset	No of subgroups	Data type	Subgroup scoring	Subgroup n
A1	3	Interval and dichotomous	Discrete scoring bands	333,333, 334
A2	3	Interval	Overlapping scoring bands	333, 333, 334
A3	6	Interval and dichotomous	Overlapping scoring bands with two distinct subgroups on each variable	166, 166, 166, 164, 168, 170
A4	3	Interval	Overlapping scoring bands plus 10 ‘noise’ variables that do not discriminate subgroups	333, 333, 334

To allow comparison across all three clustering methods, including TwoStep, these artificial datasets contained only interval +/− dichotomous data. Each dataset contained nine variables that differentiated three to six subgroups based on their scoring pattern. The complexity of the range of scores that differentiated the subgroups varied from easy (discrete and mutually exclusive scoring bands) to more difficult (overlapping scoring bands plus the presence of 10 ‘pure noise’ variables). Within each scoring band, the scores on each variable were calculated using random number generation (Excel for Mac 2008, Microsoft Corporation, Redmond, WA, USA). The sequence of individuals in the artificial datasets was randomised prior to analysis.

### Comparison criteria

The performance of the three clustering methods was compared: (i) quantitatively using the number of subgroups detected, the classification probability of individuals into subgroups and the reproducibility of results, and (ii) qualitatively using subjective judgements about each computer program’s ease of use and the ease of interpretation of the presentation of results.

The number of subgroups detected by each method was reported, along with a summary of the classification probability of each individual disc level or patient. The classification probability is an index of the certainty with which each individual was allocated into a subgroup based on their scoring pattern. For example, individuals with a scoring pattern that is stereotypical of a subgroup will be allocated with more certainty than individuals whose scoring pattern is on the boundary between two subgroups. Classification probability of individuals was not available in the TwoStep procedure.

Furthermore, the reproducibility of each method’s findings was measured by performing 10 repetitions of the clustering for each dataset. Reproducibility was reported using the number of subgroups detected, classification stability (agreement on which subgroup each individual disc-level or patient was allocated to), and classification probability (certainty of the subgroup allocation of each individual). Descriptive statistics (proportions, means, standard deviations (SD) or 95% confidence intervals (95%CI)) and trends in the number of subgroups detected were reported. Differences between classification confidence were tested using the STATA prtesti command for a one-sample test of proportions (Stata Corp, College Station, Texus, USA).

**Figure 5:**
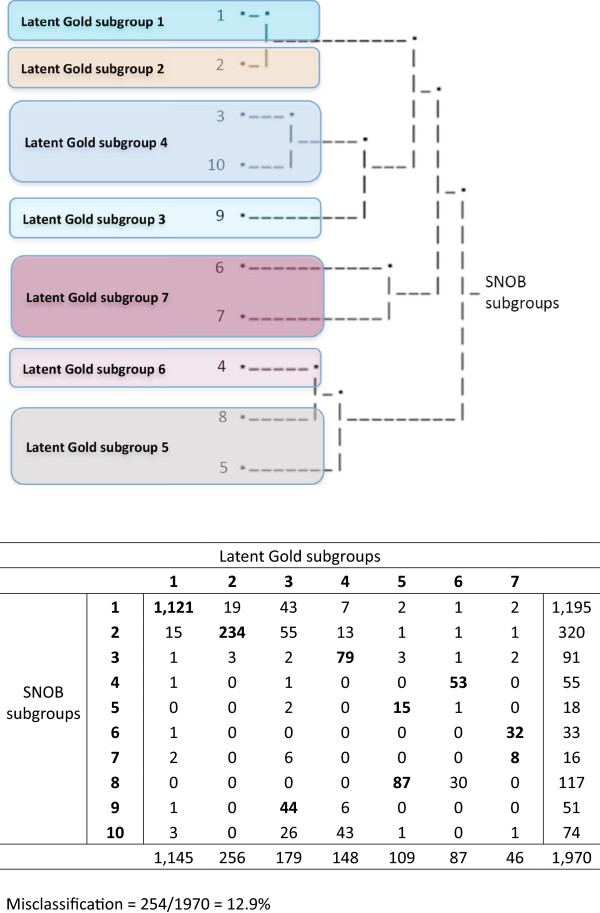
**Illustration of classification overlap of subgroups.**

Pair-wise classification disagreement between clustering methods on the allocation of individuals into subgroups was also calculated. Subgroup membership of all individuals was cross-tabulated between the final subgroup models from each clustering method, allocating each individual to the cluster in which they had the highest posterior probability. The subgroups with the highest number of individuals in these cross-tabulations were deemed to be the same subgroup and individuals classified by one method but not the other as being in that subgroup were deemed to be disagreements. The total number of pair-wise disagreements at an individual level was expressed as a proportion of the total sample size. In the case of SNOB, this process was facilitated by a tree diagram showing the derivation of the subgroups in the final model. The tree is based on a type of Bhattacharyya Coefficient that measures the similarity among subgroup probability distributions. A visual example of how this cross-tabulation was performed is shown in Figure 5.

## Results

### Real datasets

As shown in Table 3, the number of subgroups detected by each of the clustering methods varied. In every dataset, TwoStep detected the least number of subgroups, Latent Gold detected more subgroups and SNOB detected the most. This indicates that the clustering methods varied in their sensitivity to scoring patterns within the same dataset. The differences in the number of subgroups detected were typically smaller between Latent Gold and SNOB than between either of these and TwoStep, although the SMS dataset was an exception to this observation. This may have been due to a differential effect resulting from the amount of collinearity in these data, as independence of the included variables is a common assumption in clustering methods.

**Table 3:** **Classification performance with real datasets**

	TwoStep	Latent Gold	SNOB
*Number of subgroups detected*			
MRI^1^ dataset	2	7	10
MRI^2^ dataset	3	11	15
MRI^3^ dataset	2	6	7
SMS dataset	2	10	37
Clinical dataset	Not available	8	9
*Certainty (mean classification probability of disc levels or patients)*			
MRI^1^ dataset	Not available	91.2% (SD11.9%)	91.5% (11.6%)
MRI^2^ dataset	Not available	98.9% (SD3.9%)	97.1% (SD6.6%)
MRI^3^ dataset	Not available	85.7% (SD19.5%)	91.0% (SD12.7%)
SMS dataset	Not available	96.5% (SD8.8%)	98.2% (SD4.7%)
Clinical dataset	Not available	91.4% (SD12.9%)	89.9% (SD13.5%)
*Reproducibility (10 iterations of each dataset, with identical results across all datasets)*			
Number of subgroups	100% agreement	With fixed seed point = 100% agreement	100% agreement
Classification stability (reproducibility of individual disc-levels or people being classified into each subgroup)	100% agreement	With fixed seed point = 100% agreement	100% agreement
Classification certainty *(*reproducibility of the *classification probability of disc levels or patients)*	Not available	With fixed seed point = 100% agreement	100% agreement

Classification certainty (probability) was not available for TwoStep but is displayed at a group-average level in Table 3 for Latent Gold and SNOB. The standard deviation (SD) is also displayed and gives an index of the classification uncertainty that those clustering methods had in allocating individuals to subgroups. The classification certainty did not differ between Latent Gold and SNOB in the MRI^1^ dataset (p = 0.625) or the clinical dataset (p = 0.246), but it did differ in the MRI^2^ and MRI^3^ datasets and the SMS dataset (all p < 0.001). However, despite an expectation that the clustering method that was most sensitive to subgroup differences (SNOB) would also be the most certain, this was not consistently observed, as the average classification certainty was not always higher for SNOB and the differences between the methods were typically small.

**Figure 6:**
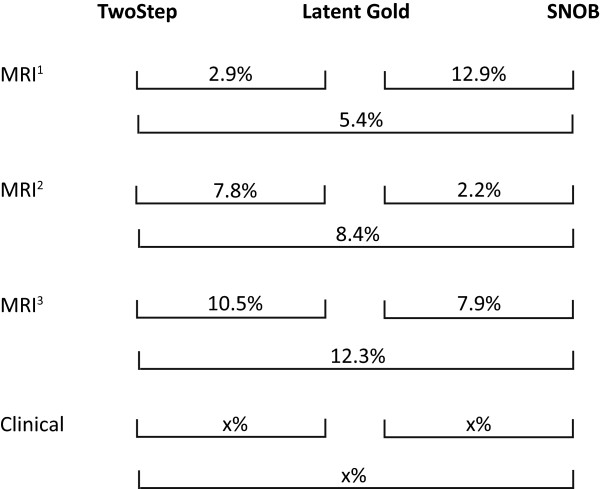
**Classification disagreement of individuals (disc levels or patients).**

The between-clustering method classification disagreement of individuals (disc levels or patients) is shown in Figure 6. The pairwise classification disagreement varied between comparisons of clustering methods, as seen by the non-overlapping confidence intervals, but there was no consistent trend that would have indicated that across datasets, some of the clustering methods more often agreed with each other.

The results for reproducibility (number of subgroups, allocation to subgroups, classification probability) are also shown in Table 3. These tests of the consistency of findings within each clustering program during 10 replications of the analysis of each dataset showed 100% agreement in all datasets and on all types of test (number of subgroups, allocation to subgroups, classification probability). In the case of Latent Gold, the default setting is to commence each analysis with a random seed point, which predictably results in some variability of the findings. As the other two clustering programs commence with a fixed but arbitrary seed point, to standardise these comparisons of reproducibility, we used a fixed but arbitrary seed point in Latent Gold in this part of the analysis.

### Artificial datasets

As shown in Table 4, these three clustering methods displayed a near-perfect ability to detect known subgroups. The only exception was that Latent Gold split one subgroup into two in artificial dataset 3 that was designed to contain 6 subgroups. We cannot rule out that our method of using random number generation to produce individual scores had produced a scoring characteristic that Latent Gold detected and used as the basis for splitting a ‘mother subgroup’ into two ‘daughter subgroups’. The classification accuracy was also very high, ranging from 98.4% to 100%.

**Table 4:** **Classification performance with artificial datasets**

	Number of subgroups detected			Accuracy of classifying 1000 individuals into subgroups		
Dataset	TwoStep	Latent Gold	SNOB	TwoStep	Latent Gold	SNOB
A1 (3 subgroups)	3	3	3	100%	100%	100%
A2 (3 subgroups)	3	3	3	99.9%	99.8%	99.9%
A3 (6 subgroups)	6	7	6	98.7%	100%	98.4%
A4 (3 subgroups)	3	3	3	99.4%	99.2%	99.4%

### Ease of use, interpretability, cost

Our subjective judgement is that these three clustering programs also varied in their ease of use and the interpretability of their presentation of results. TwoStep has the easiest learning curve, with software commands that can be all menu-driven, there is plain-language explanatory material available via the internet, the optimal subgroup solution is automatically determined, and the results are presented numerically and graphically (charts of certainty of the subgroup structure, bar and pie charts of cluster frequencies, and charts displaying the importance of specific variables to subgroups). A limitation is that Two Step is not designed to analyse ordinal data and while it is technically possible to handle such data via the use of dummy variables, this disproportionally loads the distance measure on that variable with unpredictable results on the subgroup model. As TwoStep is a component of the base module of IBM SPSS, it is available in formats that run on the IBM PC, Apple Mac and Linux platforms. Ongoing fee-based support is also available. However, the TwoStep clustering analysis component is not separately available and this software is the most expensive of these three clustering programs, usually involving annual license fees.

In our view, Latent Gold has a steeper learning curve than TwoStep, though the software commands can be menu-driven, there is abundant explanatory material and on-line training courses available, and the results are numerically and graphically presented (including a tri-plot displaying the relationships between subgroups). Latent Gold requires the analyst to determine the optimal subgroup solution but does provide a number of diagnostic measures to inform that decision and clear explanations of the relative merits of those measures. The base version of Latent Gold also allows more complex applications of LCA, such as Latent Class regression modeling and Latent Class multilevel modeling, and can also directly provide model parameters that can be used to classify new individuals who were not in the model building exercise. There is free online support for registered users and the single license fee allows perpetual use of the purchased version. A limitation is that Latent Gold is only available for the IBM PC platform.

SNOB has the steepest learning curve and is completely command line-driven in a Linux shell environment. It is the least user-friendly, requiring input data to be separated into two Linux text files, one containing the data and the other describing the variables, each with a unique syntax. The output needs to be consolidated by extracting information from the Linux shell plus information from a report file. The output is mostly numeric, although a tree diagram is produced showing the relationship between ‘mother’ and ‘daughter’ subgroups. Some explanatory material is available. This LCA program is free for not-for-profit, academic research but with minimal user support. A factor analytic version of SNOB is also available.

## Discussion

The aim of this study was to perform, using a variety of types of clinical and artificial datasets, a head-to-head comparison of three commonly available clustering methods (TwoStep, Latent Gold and SNOB). Using real clinical datasets, we found that the number of subgroups detected varied, the certainty of classifying individuals into those subgroups varied to some extent, that the findings had perfect reproducibility, that some computer programs were easier to use and that the interpretability of the presentation of findings also varied across programs. With the artificial datasets, all three clustering methods showed a near-perfect ability to detect known subgroups and correctly classify individuals into those subgroups. We believe this information will be useful to clinical researchers.

The number of subgroups detected in all the real datasets varied in a consistent pattern, with TwoStep detecting the least number of subgroups, Latent Gold detecting more subgroups and SNOB detecting the most. This variability in their sensitivity to scoring patterns within the same dataset is problematic, and in the absence of an external reference standard, it is not possible to determine what degree of sensitivity is optimal. To some extent, each clustering method may simply be reflecting the same underlying scoring structure of the data but at different levels of detail. This view appears to be supported by the results from the artificial datasets, which showed near-perfect identification of known subgroups but one instance of a subgroup being split into ‘daughter’ subgroups. Therefore, the analyst may need to choose a clustering method whose sensitivity level is appropriate for their data and the number of subgroups that are manageable and clinically meaningful. On the other hand, with all subgroup structures, it is possible to collapse subgroups together where, from a clinical perspective, there is good reason to consider them as one, or if the prevalence of a subgroup is so low as to deem it better merged with another. Therefore, an ‘overly-sensitive’ subgroup structure can be reduced by collapsing ‘daughter’ subgroups.

Eshghi et al. [29] and Gelbard et al. [13] also showed a lack of consistency across clustering techniques in the number of subgroups detected in real datasets in a comparison of diverse clustering techniques. Eshghi et al. attempted to address this lack of consistency by comparing the subgroup solutions of different clustering programs using measures of within-subgroup homogeneity and between-subgroup heterogeneity to indicate which solutions had better discrimination between subgroups. For example, to determine the within-subgroup homogeneity, they used the sum of squared deviations from the mean to compute the variation when averaged by the number of variables. Although the notion of such an external measure of discrimination is appealing, it may not be helpful in the current context. That is because clinical data is often not normally distributed, especially data collected on something other than an interval scale, and a strength of LCA techniques is their ability to model other types of data and model the probability distributions inherent in each dataset. Therefore, there is no readily apparent external reference standard by which to determine which LCA program results in the optimal subgroup solution in data when subgroup membership is unknown a priori, which is usually the case in clinical research.

Of the datasets that we analysed, only the SMS data were longitudinal. These methods of analysis did not include reference to the longitudinal time sequence inherent in these data. There are more sophisticated modeling methods available for clinical or life course trajectories that do include reference to the longitudinal nature of the data, such as latent class growth analysis and latent class growth mixture modeling [30] but comparison with these techniques was beyond the scope of our study.

Similarly, while the classification certainty (probability) varied between Latent Gold and SNOB in some datasets, this result should be interpreted with some caution and as a general guide only. That is because it was not possible to determine how comparable the measures of classification probability were between LCA programs. LCA methods may calculate classification probability using different approaches.

That there was perfect reproducibility of results (number of clusters, allocation of individuals to clusters, classification probability) is reassuring. However, analysts need to remain mindful that this perfect reproducibility is a result of the programs (except for Latent Gold) choosing an arbitrary but fixed seed point to start their analyses, and that random seed points would in some instances result in different solutions when re-running a model.

In summary, our subjective judgement is that Latent Gold offered the best balance of sensitivity to subgroups, ease of use and interpretability (Table 5). This judgement was based on its ability to manage mixed types of data, the interpretability of its findings and performance measures of subgroups, its ability to perform more complex forms of LCA, its capacity to generate model parameters that can be used to classify new individuals, and the accessibility of support. A further consideration was that while allowing analyst discretion in choosing the optimal subgroup solution might potentially introduce the capacity for bias, this process also makes explicit the criteria that were used in that choice, which may need to differ depending on the characteristics of the data and the clinical question. Compared to TwoStep, we valued the higher sensitivity, much better handling of ordinal and mixed types of data, and the more detailed output of Latent Gold. Whereas the main reason that we preferred Latent Gold over SNOB was its user-friendliness. We recognise that this judgement of the best clustering to use might vary depending on the analyst’s own expertise and support, the types of data involved, and the clinical questions to be answered.

**Table 5:** **Overall summary of three clustering techniques**

	TwoStep	Latent Gold	SNOB
Method	Distance-based, agglomerative hierarchical cluster analysis	Finite mixture modeling to probabilistically identify latent classes	Finite mixture modeling to probabilistically identify latent classes
Stopping rule to identify number of subgroups	Automated using either ‘Bayesian information criterion’ or ‘Akaike’s information criterion’	Analyst choice using various criteria, including ‘Bayesian information criterion’, unexplained variance, Chi-square p-value	Automated using ‘Minimum message length’ principle
Suitable data types	Ordinal data require recoding as dichotomous or handled as if interval data	All types	All types
Report classification probability of individuals	No	Yes	Yes
Sensitivity to subgroups	Least	Middle	Most
Reproducibility	Very high	Very high	Very high
Accuracy	Very high	Very high	Very high
Cost	Most expensive	Less expensive	Free
Support	Extensive documentation, fee-based support available	Extensive documentation and some free support available	Some documentation but minimal support available
Interpretability of presentation of results	Results are presented numerically and graphically (charts of certainty of the subgroup structure, bar and pie charts of cluster frequencies, and charts displaying the importance of specific variables to subgroups)	Results are presented numerically and graphically (including a tri-plot displaying the relationships between subgroups)	Results are mostly numeric (although a tree diagram is produced showing the relationship between ‘mother’ and ‘daughter’ subgroups)
*Learning curve (subjective judgement)*	*Easy*	*Middle*	*Hard*

A strength of this study is that it was performed using a variety of real and artificial datasets and use of a range of performance criteria. A limitation was that other LCA methods are available that we did not test, as a comprehensive comparison of all available LCA methods was beyond the scope of the study.

## Conclusions

This study compared three clustering methods (SPSS TwoStep, Latent Gold and SNOB) using a variety of datasets and performance criteria. The results from the real datasets indicated that the number of subgroups detected varied, the certainty of classifying individuals into those subgroups varied to some extent, the findings had perfect reproducibility, some programs were easier to use and the interpretability of their presentation of findings also varied across programs. The results from the artificial datasets indicated that all three clustering techniques showed a near-perfect ability to detect known subgroups and correctly classify individuals into those subgroups. Our judgement was that Latent Gold offered the best balance of sensitivity to subgroups, ease of use and interpretability but we recognise that other analysts may reach different conclusions depending on their available level of statistical support, the types of data they work with and the clinical questions they address. We believe this information will be useful to clinical researchers making decisions about which clustering methods might be appropriate to their circumstances.

## Abbreviations

BIC: Bayesian Information Criterion
GP: General medical Practitioners
LBP: Low Back Pain
LCA: Latent Class Analysis
MRI: Magnetic Resonance Imaging
SMS: Short Message Service (text messaging)
TwoStep: TwoStep Cluster Analysis in IBM SPSS.
